# The Views of the UK Public Towards Routine Neutering of Dogs and Cats

**DOI:** 10.3390/ani9040138

**Published:** 2019-04-02

**Authors:** Chanakarn Wongsaengchan, Dorothy E.F. McKeegan

**Affiliations:** Institute of Biodiversity, Animal Health & Comparative Medicine, University of Glasgow, Jarrett Building, Glasgow G61 1QH, UK; dorothy.mckeegan@glasgow.ac.uk

**Keywords:** animal ethics, animal welfare, cat, dog, neutering, public opinion, UK

## Abstract

**Simple Summary:**

While the routine neutering of pet dogs and cats is very common, it impacts on animal welfare and can be ethically problematic. Using an online and in person questionnaire, we investigated for the first time the attitudes of the UK public to the routine neutering of dogs and cats. The respondents (n = 451) expressed views both supporting and opposing routine neutering, but predominantly (>80%) supported the practice. Primary justifications were prevention of unwanted offspring and reproductive diseases. Around 10% of the respondents disagreed and felt that neutering should only be done for medical reasons. Men were less likely than women to agree with neutering of dogs and cats, and those with meat reduction diets were more likely to be against neutering. Cat owners supported neutering more than non-cat owners. Our results characterise how members of the UK public currently perceive the acceptability of routine neutering of dogs and cats.

**Abstract:**

Despite being routinely recommended by veterinarians, neutering of dogs and cats has both positive and negative impacts on animal welfare and is ethically problematic. We examined attitudes of a sample of the UK public towards routine neutering of dogs and cats using a questionnaire. Respondents indicated their level of agreement with statements describing welfare and ethical reasons ‘for’ and ‘against’ the neutering of male and female dogs and cats. We conducted a general linear model (GLM) analysis to investigate the effects of demographic factors on agreement scores. Respondents (n = 451) expressed views both supporting and opposing neutering. The predominant view (>80%) supported neutering, justified primarily by prevention of unwanted offspring and reproductive diseases. Around 10% of the respondents disagreed and felt that neutering should only be done for medical reasons. Men were less likely than women to support neutering (*p* < 0.001). Those with meat reduction diets were more likely to be against neutering (*p* < 0.05) and cat owners supported neutering more than non-cat owners (*p* < 0.05). Although the data reflected a wide range of ethical views, our findings show that the UK public generally supports the routine neutering of dogs and cats. This insight has implications for future policy-making and compliance with veterinary advice.

## 1. Introduction

Despite growing public interest in animal welfare and animal rights [1], companion animals’ reproductive life remains greatly controlled. As such, neutering dogs and cats is the most common surgical procedure carried out by veterinarians in the UK [2]. Neutering has both positive and negative impacts on animal welfare. It has been suggested that short-term negative welfare impacts include stress from unfamiliar surroundings [3], painful procedures and anaesthetic or surgical complications (8.2–9.1% of neutering cases in the UK involved one or more abnormalities) [4], while longer-term welfare impacts vary depending on the animal’s species and sex. 

Neutering is found to increase life expectancy by 13.8% in male dogs and 26.3% in female dogs [5]. In male dogs, castration removes both testes and all sex hormone effects. Thus, it is an effective treatment and prevention of androgen-induced diseases, such as benign prostatic hyperplasia (BPH), perineal hernia, prostatitis, testicular tumours and alopecia-X [6,7,8]. In addition, castration reduces the risk of infectious diseases, trauma and vascular diseases [5,9]. However, castration is reported to increase the risk of some musculoskeletal degenerative diseases (i.e., hip dysplasia, cranial cruciate rupture), obesity and associated diseases, uncommon cancers (e.g., bladder, bone, haematologic tumours), immune-mediated diseases and cognitive dysfunction syndrome [7,8,10,11,12,13,14]. While some of these are age-related diseases and were thought to be affected by the fact that neutered animals lived longer, a recent study found that neutering effects on neoplastic and immune-mediated diseases remained significant when age was controlled for and under a cumulative incidence model using competing risk data [5]. Male dogs are also castrated in an attempt to control unwanted behaviours that can reduce their welfare (i.e., frustration when exposed to oestrus females) and that cause inconvenience to their owners (i.e., escaping, mounting, urine marking and aggression) [15,16,17]. However, castration has also been found to make no difference in roaming, sexual behaviours and aggression in free-roaming dogs [18]. In fact, castrating can worsen behavioural problems in fearful dogs [19]. Thus, the likelihood of behavioural changes post-castration varies greatly depending on whether or not that behaviour is influenced by testosterone, the characteristic of the dog and its environment, the duration the dog has been showing the problematic behaviour, and whether the dog is housed or free-roaming. 

In female dogs, spaying removes both ovaries (ovariectomy—OVE) and often also the uterus (ovariohysterectomy—OVH). OVH prevents and treats potentially fatal pyometra (uterine infection), which occurs in 15.2% of bitches by four years of age and more than 50% by 10 years of age [20,21,22]. Mammary tumours are common (3.4%) and malignant in female dogs and their risk is reduced to less than 0.5% by pre-puberty neutering [23,24]. This is frequently quoted as a reason for spaying, however, a systematic review shows that its effects on mammary tumours vary from protective to no effect and age at neutering is not significant [25]. Thus, unlike pyometra, the benefits and timing of spaying to prevent mammary tumour are still controversial. The main long-term complication of spaying female dogs is urinary incontinence, particularly in large breeds and in pre-pubertal spaying, as well as obesity, cancers and other detrimental health effects as in male dogs [5,26,27,28,29]. 

Castrated male cats are reported to live, on average, 62% longer than intact male cats, and spayed female cats live a mean of 39% longer than intact female cats [30]. Castration of male cats eliminates urine odour and sometimes unwanted urine spraying and sexual behaviours as well as reducing roaming and mating fights which spread disease and cause injury [31,32,33,34,35]. In fact, intact cats are twice as likely to be infected with upper respiratory infection, 3.5 times as likely to be infected with Feline Immunodeficiency Virus (FIV), four times as likely to have ear mite infection and 4.5 times as likely to be infected with Feline Leukemia Virus (FeLV) compared to same-aged castrated and spayed cats [9]. Unlike in dogs, reproductive diseases are very rare in male cats [36]. Therefore, clinical justifications for neutering male cats are based on direct and indirect impacts on welfare and overall lower mortality rate [8,30,37]. The main drawback of neutering is an increased risk of obesity and associated diseases such as diabetes [38,39]. In female cats as in dogs, OVH prevents pyometra and mammary tumours which are more commonly malignant in cats than in dogs [24,26,40,41].

While neutering increases the lifespan overall (probably due to the reduction of fatal reproductive diseases, the absence of unwanted or risky behaviours and the reduced risks of infectious diseases), neutering appears to increase risks of several cancers and immune-mediated diseases [5,8,9]. These opposing long term welfare impacts have fuelled much debate, with regard to whether [22,42,43], how [44,45], and when to neuter dogs and cats [26,46,47,48].

Clearly, there are welfare issues associated with neutering, as well as potential societal benefits related to overpopulation control and prevention of poor welfare in unwanted offspring. Thus, neutering is also ethically problematic [42,43]. While the practice is strongly encouraged in the veterinary professional bodies in the United States of America (USA) and the United Kingdom (UK) [49,50,51], routine neutering is considered unethical by some and must be done for medical reasons in several countries such as Germany, Norway and Sweden [52,53,54,55]. In practice, the level of neutering of dogs and cats is probably highly influenced by the national veterinary associations. In the UK, policy statements issued by the British Veterinary Association (BVA) in 2006 strongly support routine neutering of cats and of female dogs but recommend that neutering of male dogs should always be considered on a case-by-case basis [50].

There are several different ethical views that attempt to define our duties to animals [56]. On a contractarian view, only the welfare of humans is concerned and neutering should be carried out if it is what the owner wants, with no regard for the animal. The utilitarian approach notes that neutering will deprive animals of pleasurable experiences such as mating and nurturing offspring [3,43,57], while not neutering intact animals raises concerns of sexual frustration of both sexes when the females are in heat [3,19]. However, it can also be argued that discomfort, hypervigilance or anxiety relating to caring for offspring, and the behavioural changes associated with heat, false pregnancy and pregnancy could be stressful for a bitch or queen as well as unpleasant for owners. Nevertheless, if neutering prevents reproductive diseases, encourages responsible pet ownership and prevents the birth of suffering offspring, the pain from the surgery and the potential loss of pleasurable experiences might be outweighed by these benefits. By contrast, those with an animal rights view may argue that neutering violates an individual animal’s right to bodily integrity, right to respectful treatment, right to perform natural behaviour, right to reproduce and right to sexuality or right not to be coercively sterilized [42,57,58,59,60]. It could be argued that on an individual basis, the welfare benefits from neutering are not so clear-cut that if healthy animals were able to choose, they would necessarily voluntarily relinquish their right to bodily integrity to for dubious future health benefits. Nevertheless, some animal rights proponents may prefer neutering over continued breeding of dependent domesticated “slave” animals [61].

To our knowledge, the attitudes of the UK public towards routine neutering of dogs and cats have never been systematically examined. Given the widespread ownership of dogs and cats (49% of UK adults) [62], this is a significant gap in knowledge. We used a questionnaire to investigate the range of views towards neutering, allowing us to determine if the predominant opinions of the public are in line with the policy statements of the veterinary associations. By determining the level of agreement with a range of statements supporting or opposing neutering, we also characterized which arguments were behind the predominant views. Finally, we also determined whether the positions adopted were related to the demographics of the respondents.

## 2. Materials and Methods

A questionnaire (full text available in Supplementary Materials) was designed to investigate the attitudes of the UK public to the routine neutering of pet dogs and cats. The study design and the questionnaire were approved by the University of Glasgow College of Medical, Veterinary & Life Sciences Ethics Committee (Application reference: 200160125). Responses were anonymous and all subjects gave their informed consent before participation. 

The first part of the questionnaire collected demographic information including gender, age, nationality, ethnicity and marital status along with eating habits, reasons for diet choice, religious beliefs and experience of dog and cat ownership. 

The second part of the questionnaire asked the participants the extent to which they agreed with a range of welfare and ethical arguments ‘for’ and ‘against’ routine neutering of dogs and cats. Male dogs, female dogs, male cats and female cats were considered separately with a similar list of statements. In each category, participants were asked to indicate their agreement with statements regarding neutering animals they hypothetically owned with responses based on a five-point Likert scale (strongly agree, agree, neutral, disagree and strongly disagree). The statements were derived from a review of literature and veterinary association policy statements [50] and were adapted according to species-specific and sex-specific costs and benefits of neutering. For example, the statements “I would neuter him to prevent or reduce unwanted behaviours.” and “I would neuter him to prevent reproductive diseases (benign prostatic hyperplasia, testicular tumour etc.)” support routine neutering in male dogs. For female dogs, these statements were changed to “I would neuter her to prevent or reduce unwanted behaviours and physiological changes (e.g., vaginal discharge)” and “I would neuter her to prevent reproductive diseases (uterine infection, breast cancer etc.)”. The respondents were provided with an open question requesting “any further comments” at the end of the questionnaire. The questionnaire also asked about attitudes to the neutering of stray/shelter dogs and cats; these data are not presented here.

The questionnaires were distributed in May–June 2017 in electronic form (via Survey Monkey) to members of the UK general public via social networking sites (Facebook). To recruit participants who do not use the internet/social media, members of the public in Glasgow were approached directly in public spaces (e.g., shopping centres, city streets, parks and squares) and were asked to fill the questionnaire in person. The minimum age of the respondents was 18 years. 

We obtained responses from 630 participants (518 from the online survey platform and 112 from the in-person interview). Only 493 responses were complete (incomplete responses were seen online only) and of these, 455 individuals identified themselves as of UK nationals. Two younger than 18-year-old respondents were excluded and two non-binary persons were omitted since the sample size of the group was not enough to be representatively analysed. Therefore, a final sample size of 451 responses was used in the data analysis. The respondents’ demographic information is shown in Table 1.

Data collected from paper questionnaires were manually entered into the online survey platform. Data in a .csv spreadsheet were then imported into R version 3.3.3 (R Core Team, 2016, http://www.R-project.org/) and analysed using general linear models (GLMs) to investigate and control for the effects of several demographic factors. Stepwise reduction of the model was conducted using Log-likelihood ratio tests (LRTs), starting from the most complex model with all possible pairwise interactions to the simplest one that explained best the response variables, using a significance level of 0.05. Each animal category (e.g., male dogs) was analysed independently. Statements were grouped ‘for’ neutering and ‘against’ neutering for each animal category. Responses of the Likert scale statements were scored as follows: strongly disagree = 1; disagree = 2; neutral = 3; agree = 4; strongly agree = 5. The scores were then averaged for the ‘for’ neutering statements and between the ‘against’ neutering statements for each animal category for each participant. These mean scores constituted the response variables, thus there were eight scores (means ‘for’ and ‘against’ neutering of each of the four animal categories: male dogs, female dogs, male cats and female cats). 

Two of the statements in the questionnaire were recognised to be problematic. “I would not neuter my animal because of religious reasons” was derived based on the Buddhism’s Five Moral Precepts. This was not relevant because the majority of the respondents were non-religious or Christian. The low scores from disagreeing to this statement could lead to misleading results for this sample; thus, this statement was omitted from the data analysis. Another specially treated statement was “I would neuter my animal only to cure a reproductive disease. Neutering should be done only for a medical reason”. This statement initially contributed to the ‘for’ score because it was supportive of neutering in certain circumstances. However, the response trend suggested that this statement was actually interpreted as an argument against neutering. In particular, people who were generally supportive of neutering showed low agreement with this statement. Therefore, we moved this statement to the ‘against’ category for analysis since not doing so inflated the ‘for’ score in participants who were against neutering. We chose not to exclude this statement since agreement levels were strong in some participants and the argument about medical reasons was an important one. A Gaussian distribution of all of the response variables was assumed in model fitting. We tested the normality of the residuals of the best-fitted models using the histogram plot and the Q-Q plot of the residuals of the selected models. Homoscedasticity (constant variance) was also examined by plotting the residuals. All of the response variables from the ‘against’ neutering statements were originally normally distributed; however, the response variables from the ‘for’ neutering statements had to be transformed prior to analysis by cubing the scores. Demographic factors (i.e., age, gender, relationship status, religious belief, diet choice and pet ownership) and their pairwise interactions were included in the most complex model as explanatory variables for each response variable. Ethnicity was not included in the analysis since 95.34% of the respondents were Caucasian (Table 1). All fixed factors and all possible interactions between them were included. The non-significant terms were removed using standard model simplification procedure via the likelihood ratio test in R, using a significance level of 0.05.

## 3. Results

### 3.1. The Predominant Views on Routine Neutering of Dogs and Cats

More than 80% of the respondents agreed with routine neutering across all categories (male dogs 88.47%; female dogs 86.03%; male cats 89.58%; female cats 87.36%), as defined by a score ‘for’ neutering over 3 and an average score ‘against’ neutering less than 3. Mean overall agreement scores supporting neutering were 4.01 ± 0.04 for male dogs, 3.94 ± 0.04 for female dogs, 4.03 ± 0.04 for male cats and 3.92 ± 0.04 for female cats. Mean overall agreement scores ‘against’ neutering were 1.98 ± 0.04 for male dogs, 1.93 ± 0.04 for female dogs, 1.88 ± 0.04 for male cats and 1.87± 0.04 for female cats. 

### 3.2. The Welfare and Ethical Reasons behind the Predominant Views

The mean agreement scores for each statement ‘for’ neutering from all participants are shown in Table 2 and mean scores for statements ‘against’ neutering are shown in Table 3. 

The highest scoring three reasons that the majority of the participants would neuter male and female dogs were “to prevent reproductive diseases”, “to prevent unwanted puppies” and “to prevent genetic disease or unwanted traits to the next generation” (Table 2). Primary reasons for neutering male and female cats were “to prevent unwanted kittens”, “to prevent reproductive diseases” and “to prevent unwanted behaviours” (Table 2). The top reasons participants would not neuter their own animals for all categories were because “neutering should be done only for a medical reason” and “the risks of the surgery”. The third ranked reason varied between categories. For male dogs, it was because “it is not necessary”, for female dogs it was because of “post-surgery inactivity, obesity and urinary incontinence” and for cats, it was related to “the pain of the surgery” (Table 3).

Free text comments regarding routine neutering were conditional upon the animal’s species, such as “Cats do tend to roam more than dogs so are better neutered”. Some reasons were also related to the animal’s sex: “Female dogs I would neuter at age 5–6 years before the risk of pyometra increases. Males I would not neuter”, “I always neuter my male cats to ensure that they do not impregnate cats where their owners are less responsible” and “Probably more important for female cats to be spayed to avoid unwanted kittens”. On the other hand, some participants suggested that sex of the animal should not matter: “I think pets regardless of gender should be treated equally”. The view that female dogs and cats should have one litter before neutering was also repeatedly presented: “I would let the females have one litter and then neuter her for her own welfare. But she should experience once an experience of mating and being a mother” and “I would allow the females to experience one litter and then I would neuter her for her own welfare”. 

Some respondents’ comments reflected animal rights views such as “I think the whole idea of domesticating animals is questionable anyway. We are equal. We are not allowed to cut any animal’s part”. However, some participants were against neutering due to welfare concerns, such as “Neutering dogs because we can is no good reason to do it. Far too many dogs are neutered far too early causing lasting damage physically and mentally” and “I will not neuter my dogs because neutering has been proven to be detrimental to temperament and health.”

Some participants emphasized that the reasons to neuter should be considered on a case by case basis: “I wouldn’t neuter my dog if he showed nervous tendencies as castration may make that behaviour worse”, “Each dog is different so in some cases it may be in the dogs best interest to NOT neuter, whether that is for behavioural or medical reasons”, “I chose neutral for some answers because I believe that those elements should be considered when letting procedure to be done with each animal”. Some neutering decisions were conditional on when and how the animal will be neutered. For example, “I would not neuter him until all his bone and joint structures were fully matured, about 18 months old” and “I would only consider neutering after sexual maturity, as scientific studies have shown it can predispose my breed to cancer”. 

The financial cost of neutering or not, was an important issue for some respondents: “It’s the costs that put people off as it’s very expensive to do”, “I have cats and as much as I would love them to have kittens I wouldn’t be able to guarantee their care if I couldn’t afford to keep them”. However, some people thought that such costs are a part of essential spending and being a responsible pet owner: “If you can’t afford vets bills then don’t have pets” and “I think it is important to take control of any over-breeding. Neutering is a small price to pay”. The pedigree status of the animal and the breeder status of the owner were also mentioned as follows: “The only other element I would consider not neutering if the animal is a pedigree”.

Some respect for nature views emerged in the comments such as “Neutering should be done to prevent overpopulation. Cat population can affect wild bird populations”, “What stops nature evolve I’m against it.” and “The survey highlights a dilemma. At which point do we stop neutering to encourage diversity and longevity of the species.”

### 3.3. Demographic Factors

**Figure 1:**
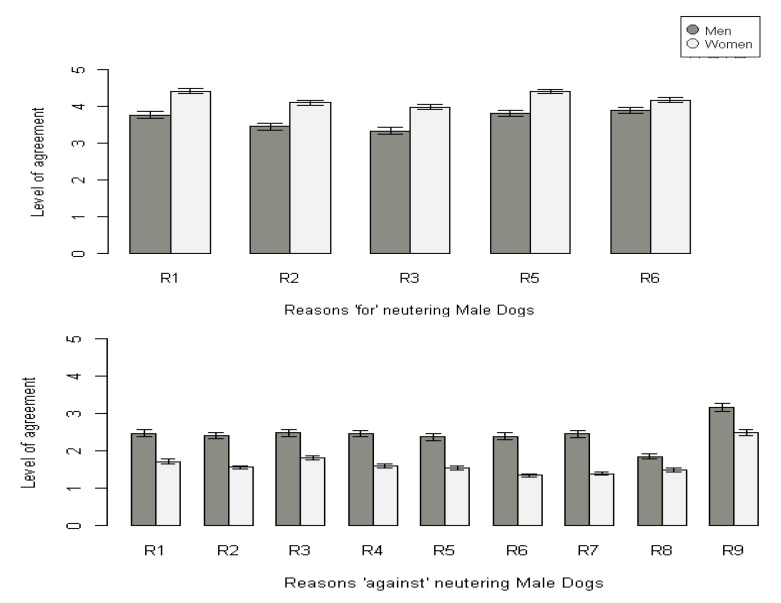
Mean agreement scores (±SE) for each statement ‘for’ and ‘against’ neutering male dogs by participant gender (men n = 161; women n = 290). ‘for’ reasons are coded as follows; R1 = To prevent unwanted puppies/kittens, R2 = To prevent unwanted behaviours, R3 = To prevent sexual frustration, R5 = To prevent reproductive diseases, R6 = To prevent unwanted genetic traits. ‘against’ reasons are coded as follows; R1 = Not necessary, R2 = The pain from the surgery, R3 = The risks of the surgery, R4 = Post-surgery inactivity, obesity or urinary incontinence, R5 = The right to body integrity, R6 = The right to reproduce, R7 = The right to have natural sexual and parental experiences, R8 = The cost, R9 = Neutering should be done only for medical reasons.

The gender of the participants affected agreement scores for male dogs, female dogs and female cats as a main effect (likelihood ratio test: 2∆LL = 60.6, df = 1, *p* < 0.001; 2∆LL = 64.4, df = 1, *p* < 0.001; 2∆LL = 48.6, df = 1, *p* < 0.001), where in all cases women were more supportive of neutering than men. For male cats there were a main effect of gender (F1,423 = 65.54, *p* < 0.001) and an interaction term with age (2∆LL = 7, df = 1, *p* < 0.05), where male participants showed less agreement with neutering and higher agreement with statements opposing neutering compared to female participants, except in older participants where this trend was reversed. Figure 1 shows data from male dogs only, illustrating that for every statement, women had higher agreement scores ‘for’ neutering than men, and men had higher ‘against’ scores than women. This pattern was apparent across all animal categories (data available in Supplementary Materials).

When it came to specific statements supporting neutering, male participants scored “to prevent genetic disease or unwanted traits to the next generation” the highest, followed by “to prevent reproductive diseases” and “to prevent unwanted puppies”. Female participants scored “to prevent unwanted puppies” the highest, followed by “to prevent reproductive diseases” and “to prevent genetic disease or unwanted traits to the next generation”.

In women, statements against neutering were ranked in line with the overall score (shown in Table 3) except for female dogs, where women scored “it is not necessary” in the third place instead of “post-surgery inactivity, obesity and urinary incontinence”. For men, the most popular reasons not to neuter animals were “neutering should be done only for a medical reason”, “the risks of the surgery” and “it is not necessary”. For male cats, “the rights to have sexual and parental experiences” as well as “it is not necessary” were ranked highest by men. The gender of participants also interacted with relationship status to affect agreement scores with statements against routine neutering of all animal categories (2∆LL = 11.14, df = 5, *p* < 0.05; 2∆LL = 15.46, df = 5, *p* < 0.01; 2∆LL = 14.42, df = 5, *p* < 0.05; 2∆LL = 14.2, df = 5, *p* < 0.05) with all women except separated women showing less agreement with statements opposing neutering female dogs than men.

Variation in agreement scores for male dogs, female dogs and male cats was affected by the respondents’ dietary choices, where respondents with a meat-reduction diet scored higher for the statements ‘against’ the routine neutering (2∆LL = 12.28, df = 4, *p* < 0.05; 2∆LL = 14.54, df = 4, *p* < 0.01; 2∆LL = 9.64, df = 4, *p* < 0.05) (Table 4).

Diet choice also affected the level of agreement with neutering of all animal categories, depending on religious belief, where vegans and vegetarians had lower agreement scores supporting neutering when they were religious (2∆LL = 19.2, df = 4, *p* < 0.001; 2∆LL = 13.2, df = 4, *p* < 0.05; 2∆LL = 14.4, df = 4, *p* < 0.01; 2∆LL = 20.6, df = 4, *p* < 0.001). Dog owners who were meat eaters, meat reduction eaters and vegan had higher agreement scores in support of neutering of male dogs (2∆LL = 11.6, df = 4, *p* < 0.05), while the opposite was true for dog owners who were vegetarian and pescetarian.

Pet ownership affected mean scores against neutering for both male and female cats as a main effect (2∆LL = 4.94, df = 1, *p* < 0.05; 2∆LL = 4.76, df = 1, *p* < 0.05), in that cat owners were less likely to be against neutering their cats than non-cat owners. Pet ownership had many interactions with other demographic factors; for example, it affected the level of agreement for neutering of all animal categories, depending on respondents’ relationship status (2∆LL = 9.6, df = 4, *p* < 0.05; 2∆LL = 10.2, df = 4, *p* < 0.05; 2∆LL = 11.4, df = 3, *p* < 0.01; 2∆LL = 18.2, df = 3, *p* < 0.001) where owners were more likely to agree with neutering depending on whether they were in a relationship. Dog owners aged between 18-29 and more than 50 years old had lower scores against neutering compared to non-dog owners (2∆LL = 7.04, df = 2, *p* < 0.05; 2∆LL = 8.78, df = 2, *p* < 0.05). Cat owners who were between 18–49 years old were more supportive of neutering than non-cat owners while those over 50 years old showed lower agreement (2∆LL = 6.4, df = 2, *p* < 0.05; 2∆LL = 13, df = 2, *p* < 0.01). 

Apart from the interaction with diet mentioned above, religious beliefs also affected the views towards neutering of both male (2∆LL = 6.8, df = 2, *p* < 0.05; 2∆LL = 12.06, df = 2, *p* < 0.01) and female dogs (2∆LL = 8,df = 2, *p* < 0.05; 2∆LL = 8.3, df = 2, *p* < 0.05), depending on respondents’ age. Opposition to neutering dogs decreased with age in non-religious participants while it was highest in 50 and older age group and lowest in the 30–49 age group in religious participants. Stacked divergent bar charts showing the percentage of responses on the Likert scale for the effects of gender on rights to sexual and parental experiences, and effects of diet choice on preventing unwanted puppies and kittens are provided in supplementary material.

## 4. Discussion

Our results indicate that more than 80% of the sampled 451 members of the UK public support the routine neutering of dogs and cats of both sexes. This reflects only a part of the policy of the BVA, which advises the routine neutering female dogs, male cats and female cats, but that neutering of male dogs should be considered on a case-by-case basis [50]. The data show that only a minority of people would not neuter their male dogs (11.53%), female dogs (13.97%), male cats (10.43%) and female cats (12.66%). These figures are lower than the estimated actual 29% of dogs not neutered currently in the UK, but slightly higher than the actual 9% of cats not neutered as reported by the PAW (People’s Dispensary for Sick Animals Animal Wellbeing) report in 2018 [62]. Views towards female dogs revealed the highest percentage of respondents who did not agree with routine neutering despite the fact that a much higher proportion of veterinary practices always recommend routine neutering of female dogs (71.3%) compared to male dogs (45%) [2]. The reasons for this finding are unclear, but may it reflect public concern that the neutering operation for females is more invasive (major surgery in the body cavity) and more prone to complications, than the male equivalent. Our results also showed that “the risks of the surgery” was the second-ranked reason opposing the spaying of female dogs. By contrast, veterinarians have better access to the information regarding the advantages and disadvantages of spaying and probably focus more on the health benefits and the reproductive diseases prevented by spaying. While the advice of UK veterinary surgeons does seem to follow the policy of the BVA, our findings suggest this is not fully reflected in the view of the UK public. 

The welfare and ethical reasons ‘for’ and ‘against’ the routine neutering of dogs and cats were very diverse. These were presented to participants, based on a review of the literature, but a free text option was also provided. While this approach was intended to gain a wider understanding of agreement with a range of arguments, we concede that some concepts or issues would not necessarily have occurred to the respondents had they not been presented. On the other hand, the free text option revealed some factors affecting the decision on neutering of the respondents that were not presented in the questionnaire such as the personality of each animal, the pedigree of the animal and the effects of the animal on wildlife. The free text comments and agreement with certain ethical arguments reflected adherence to various ethical positions including contractarian, utilitarian, animal rights, relational and respect for nature [56]. 

The primary reason that the majority of the UK public chose to support routine neutering of all animal categories (except male dogs) was, simply, to prevent unwanted offspring. For male dogs, women still gave this reason the highest score, but men had a stronger agreement with the reason ‘to prevent passing unwanted genetic traits to the next generation’. This could indicate that men were more likely to consider neutering male dogs as a case by case basis than women.

The next most popular reason in support of neutering was to prevent reproductive diseases. This argument follows utilitarian reasoning based on health benefits outweighing the welfare costs of neutering, and is very well-documented and quoted in the BVA and the BSAVA (British Small Animal Veterinary Association) policy statement [50,51]. It may reflect the fact that most owners register their dogs (90%) and cats (81%) with veterinary practices and may have been given this information [62,63]. However, while neutering as a prophylactic treatment of reproductive diseases is a significant benefit in male dogs, female dogs and female cats, it is not really relevant to male cats where reproductive diseases are very rare [8]. Since this reason had similarly high scores across all animal categories, it is important that the public is correctly informed before assuming there are health benefits that arise from neutering.

Interestingly, the third-ranked reason ‘for’ neutering was different between dogs and cats. For dogs, it was based on preventing perpetuation of unwanted genetic traits; however, for cats, it was about preventing unwanted behaviours. This presumably reflects the fact that the control of hereditary diseases is more important in dogs than in cats since there are approximately 900 hereditary disorders and genetic predispositions in dogs and only about 200 in cats [64]. Exaggerated conformation in pedigree dogs was reported among the three most concerning problems among UK vets in the PAW report [65], but it was not a concern in cats. Our results probably also reflect that neutering is more commonly used as an effective solution to control unwanted behaviours in cats than in dogs since roaming, fighting and spraying in cats are problematic as well as hormonally influenced [51,66]. Although neutering carries less risk of complications in cats than in dogs [4,67], the risk of mortality in cats is higher primarily due to the risk of anaesthetic complications [68]. Cats were also more likely than dogs to be neutered: 91.7% versus 78.5% and 60% versus 26.4% in a previous study in New Zealand and the USA, respectively [69,70], suggesting that owners might favour neutering cats to prevent unwanted behaviour and/or might not be aware that there is a greater risk of mortality but not of complications in cats compared to dogs. However, another study showed that there was little difference between the percentage of owners in the UK who wanted to change at least one behaviour of their dogs (78%) and their cats (77%) [62].

Apart from the arguments on whether to neuter companion animals, when (e.g., prepubertal, after one heat, after one litter) and how (e.g., OVE or OVH) to neuter have also been controversial [22,26,44,47,48]. In this study, we did not focus on the details of when and how neutering should be done, but the belief that female animals should have one litter before being spayed was apparent in the free text comments. While 91.2% of veterinary practices in the UK reported that they would never recommend this, 9.6% of dog owners spayed their bitches after one litter of puppies [2], and almost half of the cat owners (49%) believed a female cat should have a litter before being neutered [71]. This misapprehension could contribute to population growth and the number of unwanted animals entering animal welfare organisations and shelters.

Although being ‘against’ neutering was a minority view in this study, participants agreed with a range of welfare and ethical reasons not to neuter dogs and cats. One was that neutering should be done only for medical reasons. Utilitarians and even animal rights supporters would agree with this as compulsory and vital to the animal’s life and well-being. Since it scored higher than other reasons, the argument that neutering should only be done for medical reasons or on a case-by-case basis for each individual animal could gain traction in the UK, as already practised in some countries [52,53,54,55]. Another important reason opposing routine neutering in every animal category was because of the risk of the surgery itself. Even though complications from neutering are reported to be rare and mild [4,22,67], some participants did not think it was worth the risk. However, in a recent study in Ireland, where 43 pet owners were interviewed with an open question “What influenced your decision to have your pet neutered or not?”, the reason of being afraid that animals could die from the surgery was not reported [72].

The other welfare reasons against neutering varied according to the animal’s species, sex and whether the respondents were men or women. There was more concern for cats regarding pain from the surgery than dogs. This result is in contrast with a study reporting that routine neutering surgeries were considered by veterinarians to be more painful in dogs than in cats [73]. This may due to the different perceptions between these stakeholders in that the veterinarians considered that they performed a more invasive procedure on dogs which are mostly larger in terms of body, organs and incision wounds than cats. However, owners may have based their responses on the perception that cats were more prone to post-operative stress than dogs, based on their behaviour.

Respondents were more concerned about obesity and urinary incontinence after neutering female animals than obesity after neutering male animals. This could be because the female scenario stated in the questionnaire sounded more serious for having both illnesses compared to one in males. However, it could also be because obesity alone was underestimated by pet owners in its potential to harm the welfare of their animals. Obesity occurs commonly in both sexes after neutering and has always been among the top welfare issues concerning veterinarians [2,38,62].

Another key finding in the present study was that demographic factors affected attitudes towards neutering. The gender of the respondents was a highly significant factor, where men were less likely to neuter dogs and cats than women in this study. The same finding was reported in previous studies in Australia and New Zealand [69,74]. Although there was a gender bias toward female respondents in this survey (64.3% women as opposed to 35.7% men), the results are still applicable, because there appear to be more female owners than male owners of dogs (54% versus 46%) and cats (58% versus 42%) in the UK [65]. In addition, regardless of ownership, women may be more likely to be primary caregivers of pets with a decision-making role, and thus, their views are highly relevant [75]. Across all animal categories, men showed higher levels of agreement than women that depriving animals of sexual and parental experiences was a valid argument against neutering. This concurs with a previous study in Australia and New Zealand, where approximately twice as many male owners agreed that castrating male dogs removed ‘maleness’ and also more men than woman agreed with the question; “Do you equate dog sexuality with human sexuality?” [69,74]. However, this does not explain why men are less supportive than women of neutering female cats and dogs. In a study of cat neutering, men were more than twice as likely as women to believe that a female cat should have a litter before being neutered [71]. Men were also more likely than women to agree with the statement; “Cats and dogs have the right to remain whole and have offspring” (24% versus 9%) and the statement; “Sterilization removes the sexuality/masculinity of the cat or dog” (29.1% versus 13.4%) [69,76]. Therefore, the observed gender differences may reflect knowledge, favoured animal rights views and perceptions in men that pet personality is related to gonadal status [69,71,76]

We expected agreement with arguments based on rights to body integrity, to reproduce and to have sexual and parental experiences to be more common in the present study. In addition, we anticipated that the financial cost would be important. Though these two arguments did not feature strongly in agreement ratings from the questionnaire, they were emphasised in some of the comments. This concurs with pet owners in other countries who contend that dogs and cats have the right to remain whole and have offspring and that the cost of neutering stops people from neutering [69,70]. In the UK, only the cost aspect has been studied among pet owners, where 20.1% of the owners surveyed had their dogs neutered in a free or subsidized neutering scheme [2].

Diet choice also affected the responses in this study. We expected vegetarians and vegans to be more likely to hold animal rights views than conscientious omnivores [77], however, meat reduction eaters had the highest agreement scores ‘against’ neutering. Surprisingly, vegans had the highest ‘for’ scores, perhaps because they are particularly interested in animal welfare, and therefore more supportive of neutering to prevent the suffering of unwanted offspring. Importantly, unlike Lund et al. (2016), we were specifically determining the perceptions of the respondents towards companion animals as opposed to animals for consumption, which may have affected the results. Vegetarians and vegans in the present study scored lower on the ‘for’ neutering statements when they were religious, compared to respondents who had no religion. Most religious respondents were Christian, and depending on their interpretation of the Bible, they may view neutering is an acceptable act of domination over animals, a caring responsibility or cruelty and sign of ingratitude to the Creator [78].

Another demographic factor influencing attitudes towards neutering was the experience of pet ownership. Cat owners were less likely to be ‘against’ neutering their cats than non-cat owners. This is understandable as cats were seen to be more likely to roam than dogs and neutering is an effective means to solve this and other behavioural problems in cats [43,51,72]. In fact, there was a much greater proportion of current or previous dog (87.93%) and cat (83.10%) owners compared to non-owners in this study. The reasons for this are unclear but probably reflect recruitment bias in that current or previous pet owners were more likely to be interested in completing the questionnaire. Arguably, however, the views of previous or current pet owners are most relevant to the examination of this issue so this could be considered to be a strength of the current study. 

Noncompletion of the questionnaire was seen only in online participants, probably due to the length of the questionnaire and the repetitive questions in different animal categories. It is not clear if the exclusion of incomplete surveys affected the results. It could have introduced bias via demographics towards people who are more interested in the topic. While recruitment via social media always risks recruitment bias, we attempted to mitigate this with additional in-person questionnaire completion. In fact, our age, ethnicity, religion and diet choice demographic profile (Table 1) reasonably matches that of the UK population: the biggest age group (40.4%) is the prime working age (25–54 years), 87.5% of the UK population are Caucasian, more than half (53%) of the UK public are non-religious and 33.5% of the population are cutting down or cutting out meat [79,80,81,82]. We controlled for these demographic factors as well as gender, relationship status and pet ownership in our analysis. Level of education was not determined, and while some participants appeared to be very knowledgeable, others commented that they had not thought about the reasons ‘for’ and ‘against’ neutering before. While we acknowledge the limitations of this opportunistic sample and the need for caution with generalisation, this study nevertheless provides the first data on public attitudes to neutering in the UK.

## 5. Conclusions

The respondents in this study expressed agreement with statements both supporting and opposing routine neutering of dogs and cats. Strong support of routine neutering was clear, showing that this convenience sample of the UK public is generally in favour of routine neutering, as recommended by the veterinary profession (e.g., BVA), but apparently are not aware of the caveat that neutering male dogs should be considered on a case-by-case basis. It is not clear to what extent these public attitudes have been shaped by veterinary advice; however, its influence is likely to be significant. Although some respondents were very well informed, some misunderstandings regarding the welfare costs and benefits of neutering were apparent, some of which have the potential to contribute to pet overpopulation. Animal rights opposition to neutering based on bodily integrity and rights to reproduce were less pronounced than we expected, even among respondents with vegetarian and vegan diets. These data improve our understanding of current attitudes towards an important animal welfare issue and highlight factors that influence views towards routine neutering, most notably gender, and provide a basis for comparison with other countries. Rollin [53] noted that Europeans are generally against routine neutering while in the USA, pet owners view it as a moral obligation. Our findings indicate that the UK public support the routine neutering of all animal categories. which seems to be more in line with the perspective in the USA than some other European countries [52,54,55]. This leads to the intriguing question of why these differences in attitudes exist which is an area for further study. The findings also identify areas of focus for improved communication with pet owners to protect animal welfare, improve compliance with veterinary advice and reduce the risk of pet overpopulation.

## Figures and Tables

**Table 1:** Descriptive statistics regarding the age, gender, relationship status, ethnicity, religious beliefs, dietary habits and pet ownership experience of the respondents (n and percentage).

Demographic Factors	Female	%	Male	%	Total	%
	(n = 290)		(n = 161)		(n = 451)	
**Age**						
18-29	66	22.75%	61	37.89%	127	28.16%
30-49	131	45.17%	64	39.75%	195	43.24%
50 and older	93	32.07%	36	22.36%	129	28.60%
**Relationship Status**						
Married	120	41.38%	38	23.60%	158	35.03%
Single	72	24.83%	89	55.28%	161	35.70%
Living with partner	64	22.07%	23	14.29%	87	19.29%
Divorced	19	6.55%	7	4.35%	26	5.76%
Widowed	9	3.10%	3	1.86%	12	2.66%
Separated	6	2.07%	1	0.62%	7	1.55%
**Ethnicity**						
Caucasian	278	95.86%	152	94.41%	430	95.34%
Multiple ethnicity	10	3.45%	3	1.86%	13	2.88%
Other	2	0.68%	6	3.73%	8	1.77%
**Religion**						
Non-religious	197	67.93%	108	67.08%	305	67.63%
Religious *	93	32.07%	53	32.92%	146	32.37%
**Dietary Habits**						
Meat-eater	176	60.69%	126	78.26%	302	66.96%
Meat reduction	44	15.17%	20	12.42%	64	14.19%
Pescetarian	18	6.21%	5	3.11%	23	5.10%
Vegetarian	24	8.28%	8	4.97%	32	7.10%
Vegan	28	9.66%	2	1.24%	30	6.65%
**Current/previous dog owner**	255	87.93%	108	67.08%	363	80.49%
**Current/previous cat owner**	241	83.10%	104	64.60%	345	76.50%

* Religious affiliations: Christian (95.57%), Jewish (0.22%), Hinduism (0.22%), Pecan (1.33%), Spiritualist (0.67%), Agnostic (0.67%) and other/not stated (1.33%). The demographic factors asked in the questionnaire are in bold.

**Table 2:** Mean (±SD) agreement scores (based on assigning strongly disagree = 1; disagree = 2; neutral = 3; agree = 4; strongly agree = 5) for each statement supporting the routine neutering of each animal category from all participants (n = 451).

Reasons ‘For’ Neutering to Prevent:	Male Dogs	Female Dogs	Male Cats	Female Cats
Unwanted puppies/kittens	4.18 ± 1.13	4.25 ± 0.93	4.37 ± 0.97	4.42 ± 0.93
Unwanted behaviours	3.87 ± 1.13	3.88 ± 1.14	4.23 ± 1.08	4.02 ± 1.14
Sexual frustration	3.75 ± 1.23	3.64 ± 1.26	3.83 ± 1.23	3.82 ± 1.26
Pregnancy discomfort and pain	-	3.54 ± 1.28	-	3.74 ± 1.28
Reproductive diseases	4.20 ± 1.05	4.24 ± 0.93	4.35 ± 0.91	4.31 ± 0.93
Unwanted genetic traits	4.07 ± 1.19	4.06 ± 1.53	3.38 ± 1.51	3.25 ± 1.53

**Table 3:** Mean (±SD) agreement scores (based on assigning strongly disagree = 1; disagree = 2; neutral = 3; agree = 4; strongly agree = 5) for each statement opposing the routine neutering of each animal category from all participants (n = 451).

Reasons ‘Against’ Neutering	Male Dogs	Female Dogs	Male Cats	Female Cats
Not necessary	1.98 ± 1.13	1.85 ± 1.18	1.74 ± 1.06	1.69 ± 1.02
The pain from the surgery	1.86 ± 1.04	1.89 ± 1.03	1.77 ± 1.01	1.81 ± 1.01
The risks of the surgery	2.05 ± 1.12	2.02 ± 1.09	1.86 ± 1.05	1.93 ± 1.09
Post-surgery inactivity, obesity or urinary incontinence	1.91 ± 1.05	1.89 ± 1.06	1.76 ± 0.98	1.77 ± 1.01
The right to body integrity	1.83 ± 1.11	2.00 ± 1.19	1.75 ± 1.06	1.75 ± 1.04
The right to reproduce	1.72 ± 1.02	1.74 ± 1.21	2.00 ± 1.01	1.71 ± 1.02
The right to have natural sexual and parental experiences	1.77 ± 1.07	1.74 ± 1.06	1.73 ± 1.05	1.70 ± 1.02
The cost of neutering	1.62 ± 0.93	1.63 ± 0.94	1.60 ± 0.92	1.60 ± 0.93
Neutering should be done only for medical reasons	2.73 ± 1.42	2.84 ± 1.44	3.00 ± 1.49	2.90 ± 1.46

**Table 4:** Mean agreement scores (± SD) for participants by diet choice (meat (n = 176), meat reduction (n = 44), pescetarian (n = 18), vegetarian (n = 24) and vegan (n = 28)) supporting or opposing routine neutering of each animal category.

Diet Choice	‘For‘ Neutering				‘Against‘ Neutering			
	Male Dogs	Female Dogs	Male Cats	Female Cats	Male Dogs	Female Dogs	Male Cats	Female Cats
Meat	4.04 ± 0.84	3.95 ± 0.87	4.03 ± 0.74	3.92 ± 0.78	1.96 ± 0.85	1.91 ± 0.81	1.87 ± 0.79	1.87 ± 0.81
Meat reduction	3.98 ± 0.74	3.90 ± 0.82	3.98 ± 0.70	3.88 ± 0.73	2.16 ± 0.98	2.14 ± 0.95	2.07 ± 0.93	2.07 ± 0.95
Pescetarian	3.89 ± 1.14	3.78 ± 1.20	4.09 ± 0.83	3.91 ± 0.90	1.98 ± 0.98	1.91 ± 0.92	1.76 ± 0.91	1.73 ± 0.83
Vegetarian	3.69 ± 1.10	3.83 ± 1.04	3.97 ± 0.89	3.93 ± 0.95	2.03 ± 1.04	1.88 ± 0.93	1.87 ± 0.93	1.84 ± 0.93
Vegan	4.24 ± 0.95	4.12 ± 0.95	4.13 ± 0.76	4.06 ± 0.70	1.81 ± 0.75	1.73 ± 0.69	1.60 ± 0.62	1.60 ± 0.64

## Supplementary Materials

The following are available online at https://www.mdpi.com/2076-2615/9/4/138/s1. The full text of the questionnaire and additional figures are available as supplementary materials.

## References

[1] Lundmark F., Berg C., Schmid O., Behdadi D., Röcklinsberg H. (2014). Intentions and Values in Animal Welfare Legislation and Standards. J. Agric. Environ. Eth..

[2] Diesel G., Brodbelt D., Laurence C. (2010). Survey of veterinary practice policies and opinions on neutering dogs. Vet. Rec..

[3] Sandøe P., Corr S., Palmer C. (2016). Routine Neutering of Companion Animals. Companion Animal Ethics.

[4] (2018). In brief: Complication rates of neutering revealed. Vet. Rec..

[5] Hoffman J.M., Creevy K.E., Promislow D.E.L. (2013). Reproductive Capability Is Associated with Lifespan and Cause of Death in Companion Dogs. PLoS ONE.

[6] Rosser E.J., von Tscharner C., Halliwell R.E.W. (1990). Castration-responsive dermatosis in the dog. Advances in Veterinary Dermatology.

[7] Teske E., Naan E.C., Van Dijk E.M., Van Garderen E., Schalken J.A. (2002). Canine prostate carcinoma: Epidemiological evidence of an increased risk in castrated dogs. Mol. Cell. Endocrinol..

[8] Reichler I. (2009). Gonadectomy in Cats and Dogs: A Review of Risks and Benefits. Reprod. Domest. Anim..

[9] Banfield Pet Hospital State of Pet Health 2014 Report. https://www.banfield.com/Banfield/media/PDF/Downloads/soph/Banfield-State-of-Pet-Health-Report_2014.pdf.

[10] Hart B.L. (2001). Effect of gonadectomy on subsequent development of age-related cognitive impairment in dogs. J. Am. Vet. Med. Assoc..

[11] Fulkerson C.M., Dhawan D., Ratliff T.L., Hahn N.M., Knapp D.W. (2017). Naturally Occurring Canine Invasive Urinary Bladder Cancer: A Complementary Animal Model to Improve the Success Rate in Human Clinical Trials of New Cancer Drugs. Int. J. Genomics.

[12] Hart B.L., Hart L.A., Thigpen A.P., Willits N.H. (2014). Long-Term Health Effects of Neutering Dogs: Comparison of Labrador Retrievers with Golden Retrievers. PLoS ONE.

[13] Cooley D.M., Beranek B.C., Schlittler D.L., Glickman N.W., Glickman L.T., Waters D.J. (2002). Endogenous gonadal hormone exposure and bone sarcoma risk. Cancer Epidemiol. Biomark. Prev..

[14] Polisca A., Troisi A., Fontaine E., Menchetti L., Fontbonne A. (2016). A retrospective study of canine prostatic diseases from 2002 to 2009 at the Alfort Veterinary College in France. Theriogenology.

[15] Hopkins S.G., Schubert T.A., Hart B.L. (1976). Castration of adult male dogs: Effects on roaming, aggression, urine marking, and mounting. J. Am. Vet. Med. Assoc..

[16] Serpell J.A., Hsu Y.A. (2005). Effects of breed, sex, and neuter status on trainability in dogs. Anthrozoos.

[17] Mat Ward APBC|Neutering Male Dogs—The Behavioural Effects of Castration. https://www.apbc.org.uk/pet-owner-article/neutering-male-dogs-the-behavioural-effects-of-castration/.

[18] Garde E., Pérez G.E., Vanderstichel R., Dalla Villa P.F., Serpell J.A. (2016). Effects of surgical and chemical sterilization on the behavior of free-roaming male dogs in Puerto Natales, Chile. Prev. Vet. Med..

[19] Warnes C. Castration Risks and Benefits: Dogs. https://www.apbc.org.uk/wp-content/uploads/2018/10/apbc_summary_sheet_of_castration_risks_and_benefits.pdf.

[20] Fukuda S. (2001). Incidence of Pyometra in Colony-raised Beagle Dogs. Exp. Anim..

[21] Jitpean S., Hagman R., Ström Holst B., Höglund O., Pettersson A., Egenvall A. (2012). Breed Variations in the Incidence of Pyometra and Mammary Tumours in Swedish Dogs. Reprod. Domest. Anim..

[22] Romagnoli S. Surgical gonadectomy in the bitch and queen: Should it be done and at what age?. Proceedings of the Southern European Veterinary Conference and Congreso Nacional AVEPA.

[23] Robbins M., Slatter D.H. (2003). Reproductive oncology. Textbook of Small Animal Surgery.

[24] Schneider R., Dorn C.R., Taylor D.O. (1969). Factors influencing canine mammary cancer development and postsurgical survival. J. Natl. Cancer Inst..

[25] Beauvais W., Cardwell J.M., Brodbelt D.C. (2012). The effect of neutering on the risk of mammary tumours in dogs—A systematic review. J. Small Anim. Pract..

[26] Root Kustritz M.V. (2018). Population Control in Small Animals. Vet. Clin. N. Am. Small Anim. Pract..

[27] Thrusfield M.V., Holt P.E., Muirhead R.H. (1998). Acquired urinary incontinence in bitches: Its incidence and relationship to neutering practices. J. Small Anim. Pract..

[28] Angioletti A., De Francesco I., Vergottini M., Battocchio M.L. (2004). Urinary Incontinence After Spaying in the Bitch: Incidence and Oestrogen-therapy. Vet. Res. Commun..

[29] David G., Rajendran E.I. (1980). The after-effects of spaying in bitches and cats. Cheiron Tamil Nadu J. Vet. Sci. Anim. Husb..

[30] Banfield Pet Hospital State of Pet Health 2013 Report. https://www.banfield.com/Banfield/media/PDF/Downloads/soph/Banfield-State-of-Pet-Health-Report_2013.pdf.

[31] Hart B.L., Cooper L. (1984). Factors relating to urine spraying and fighting in prepubertally gonadectomized cats. J. Am. Vet. Med. Assoc..

[32] Knol B.W., Egberink-Alink S.T. (1989). Treatment of problem behaviour in dogs and cats by castration and progestagen administration: A review. Vet. Q..

[33] Hendriks W.H., Rutherfurd-Markwick K.J., Weidgraaf K., Ugarte C., Rogers Q.R. (2008). Testosterone increases urinary free felinine, N-acetylfelinine and methylbutanolglutathione excretion in cats (Felis catus). J. Anim. Physiol. Anim. Nutr. (Berl.).

[34] Tarttelin M.F., Hendriks W.H., Moughan P.J. (1998). Relationship between plasma testosterone and urinary felinine in the growing kitten. Physiol. Behav..

[35] Spain C.V., Scarlett J.M., Houpt K.A. (2004). Long-term risks and benefits of early-age gonadectomy in cats. J. Am. Vet. Med. Assoc..

[36] Yamamoto J.K., Hansen H., Ho E.W., Morishita T.Y., Okuda T., Sawa T.R., Nakamura R.M., Pedersen N.C. (1989). Epidemiologic and clinical aspects of feline immunodeficiency virus infection in cats from the continental United States and Canada and possible mode of transmission. J. Am. Vet. Med. Assoc..

[37] Beate Kalz D.-B., Berlin I., Mlynek J., Ronacher Gutachter B., Elepfandt A., Scheibe S.K.M., Juhr N.C. (2001). Populationsbiologie, Raumnutzung und Verhalten verwilderter Hauskatzen und der Effekt von Maßnahmen zur Reproduktionskontrolle.

[38] Nguyen P.G., Dumon H.J., Siliart B.S., Martin L.J., Sergheraert R., Biourge V.C. (2004). Effects of dietary fat and energy on body weight and composition after gonadectomy in cats. Am. J. Vet. Res..

[39] Prahl A., Guptill L., Glickman N.W., Tetrick M., Glickman L.T. (2007). Time trends and risk factors for diabetes mellitus in cats presented to veterinary teaching hospitals. J. Feline Med. Surg..

[40] Potter K., Hancock D.H., Gallina A.M. (1991). Clinical and pathologic features of endometrial hyperplasia, pyometra, and endometritis in cats: 79 cases (1980–1985). J. Am. Vet. Med. Assoc..

[41] Overley B., Shofer F.S., Goldschmidt M.H., Sherer D., Sorenmo K.U. (2005). Association between Ovarihysterectomy and Feline Mammary Carcinoma. J. Vet. Intern. Med..

[42] Boonin D. (2003). Robbing PETA to Spay Paul: Do Animal Rights Include Reproductive Rights?. Between Species An Online J. Study Philos. Anim..

[43] Palmer C., Corr S., Sandøe P. (2012). Inconvenient Desires: Should We Routinely Neuter Companion Animals?. Anthrozoos A Multidiscip. J. Interact. People Anim..

[44] Van Goethem B., Schaefers-okkens A., Kirpensteijn J. (2006). Making a Rational Choice Between Ovariectomy and Ovariohysterectomy in the Dog: A Discussion of the Benefits of Either Technique. Vet. Surg..

[45] Peeters M.E., Kirpensteijn J. (2011). Comparison of surgical variables & short-term post-op complications in healthy dogs undergoing OVH or OVE. J. Am. Vet. Med. Assoc..

[46] Howe L. (2015). Current perspectives on the optimal age to spay/castrate dogs and cats. Vet. Med. Res. Reports.

[47] Clark K. (2012). Neutering: How early is too early?. Vet. Rec..

[48] Farnworth M.J., Adams N.J., Seksel K., Waran N.K., Beausoleil N.J., Stafford K.J. (2013). Veterinary attitudes towards pre-pubertal gonadectomy of cats: A comparison of samples from New Zealand, Australia and the United Kingdom. N. Z. Vet. J..

[49] AVMA (American Veterinary Medical Association) Spaying & Neutering. https://ebusiness.avma.org/files/productdownloads/spay_neuter_brochure.pdf.

[50] BVA (British Veterinary Association) BVA Policy—Neutering of Cats and Dogs. https://www.bva.co.uk/News-campaigns-and-policy/Policy/Companion-animals/Neutering/.

[51] BSAVA (British Small Animal Veterinary Association) Position Statements: Neutering. https://www.bsava.com/Resources/Veterinary-resources/Position-statements/Neutering.

[52] Günzel-Apel A.R. (1998). Early castration of dogs and cats from the point of view of animal welfare. Dtsch. Tierarztl. Wochenschr..

[53] Rollin B. (2003). Oncology and Ethics. Reprod. Domest. Anim..

[54] Paige M. Tomaselli Detailed Discussion of International Comparative Animal Cruelty Laws|Animal Legal & Historical Center. https://www.animallaw.info/article/detailed-discussion-international-comparative-animal-cruelty-laws.

[55] SVS (Sveriges Veterinärförbund) Norm angående kirurgisk kastration av friska Hundar [Norm Regarding the Surgical Neutering of Healthy Dogs]. http://www.svf.se/Documents/S%C3%A4llskapet/Veterin%C3%A4rkongressen/Kompendiet/Veterin%C3%A4rkongressen%202012%20webb%20(2).pdf.

[56] Sandøe P., Christiansen S.B. (2008). What are our duties to animals?. Ethics of Animal Use.

[57] Palmer C., Pedersen H.G., Sandøe P. (2018). Beyond Castration and Culling: Should We Use Non-surgical, Pharmacological Methods to Control the Sexual Behavior and Reproduction of Animals?. J. Agric. Environ. Eth..

[58] Singer P. (1975). Animal Liberation: A New Ethics for Our Treatment of Animals.

[59] Davison-Vecchione D., Pambos K., Steven M. (2017). Wise and the Common Law Case for Animal Rights: Full Steam Ahead. Can. J. Law Jurisprud..

[60] Taylor A. (2014). An Interview with Sue Donaldson and Will Kymlicka. Between Species.

[61] Gary L. Francione Animal Rights and Domesticated Nonhumans—Animal Rights the Abolitionist Approach. https://www.abolitionistapproach.com/animal-rights-and-domesticated-nonhumans/.

[62] PDSA (People’s Dispensary for Sick Animals) PDSA Animal Wellbeing (PAW) Report. https://www.pdsa.org.uk/media/4372/paw-2018-full-web-ready-a4-printable.pdf.

[63] Murray J.K., Roberts M.A., Whitmarsh A., Gruffydd-Jones T.J. (2009). Survey of the characteristics of cats owned by households in the UK and factors affecting their neutered status. Vet. Rec..

[64] Urs Giger Managing and Controlling Hereditary Disease. http://veterinarycalendar.dvm360.com/managing-and-controlling-hereditary-disease-proceedings.

[65] PDSA (People’s Dispensary for Sick Animals) PDSA Animal Wellbeing (PAW) Report. https://www.pdsa.org.uk/media/3290/pdsa-paw-report-2017_online-3.pdf.

[66] The Cat Group Policy Statement 1: Timing of Neutering. http://www.thecatgroup.org.uk/policy_statements/neut.html.

[67] Pollari F.L., Bonnett B.N., Bamsey S.C., Meek A.H., Allen D.G. (1996). Postoperative complications of elective surgeries in dogs and cats determined by examining electronic and paper medical records. J. Am. Vet. Med. Assoc..

[68] Brodbelt D. (2009). Perioperative mortality in small animal anaesthesia. Vet. J..

[69] McKay S.A., Farnworth M.J., Waran N.K. (2009). Current Attitudes toward, and Incidence of, Sterilization of Cats and Dogs by Caregivers (Owners) in Auckland, New Zealand. J. Appl. Anim. Welf. Sci..

[70] Faver C.A. (2009). Sterilization of Companion Animals: Exploring the Attitudes and Behaviors of Latino Students in South Texas. J. Appl. Anim. Welf. Sci..

[71] Welsh C.P., Gruffydd-Jones T.J., Roberts M.A., Murray J.K. (2014). Poor owner knowledge of feline reproduction contributes to the high proportion of accidental litters born to UK pet cats. Vet. Rec..

[72] Downes M.J., Devitt C., Downes M.T., More S.J. (2015). Neutering of cats and dogs in Ireland; pet owner self-reported perceptions of enabling and disabling factors in the decision to neuter. PeerJ.

[73] Kongara K., Squance H., Topham I., Bridges J. (2016). Attitudes and perceptions of veterinary paraprofessionals in New Zealand to postoperative pain in dogs and cats. N. Z. Vet. J..

[74] Blackshaw J., Day C. (1994). Attitudes of dog owners to neutering pets: Demographic data and effects of owner attitudes. Aust. Vet. J..

[75] Westgarth C., Pinchbeck G.L., Bradshaw J.W.S., Dawson S., Gaskell R.M., Christley R.M. (2010). Factors associated with cat ownership in a community in the UK. Vet. Rec..

[76] Nicole Wilde Why Do So Many Men Say Nuts to Neutering?. https://www.dogstardaily.com/blogs/why-do-so-many-men-say-nuts-neutering.

[77] Lund T.B., McKeegan D.E.F., Cribbin C., Sandøe P. (2016). Animal Ethics Profiling of Vegetarians, Vegans and Meat-Eaters. Anthrozoos.

[78] Bekoff M. (2010). Religion and animals. Encyclopedia of Animal Rights and Animal Welfare.

[79] Central Intelligence agency United Kingdom Demographics Profile 2018. https://www.indexmundi.com/united_kingdom/demographics_profile.html.

[80] Office for National Statistics Overview of the UK Population. https://www.ons.gov.uk/peoplepopulationandcommunity/populationandmigration/populationestimates/articles/overviewoftheukpopulation/november2018.

[81] NatCen British Social Attitudes Survey: Religious Affiliation among Adults in Great Britain. http://www.natcen.ac.uk/media/1469605/BSA-religion.pdf.

[82] Waitrose Food and Drink Report 2018–2019. https://www.waitrose.com/content/dam/waitrose/Inspiration/Waitrose&PartnersFoodandDrinkReport2018.pdf.

